# Statistical analysis plan for the LAST ACT clinical trial; a Leukotriene A4 hydrolase Stratified non-inferiority Trial of Adjunctive Corticosteroids for HIV-negative adults with Tuberculous meningitis

**DOI:** 10.12688/wellcomeopenres.22498.1

**Published:** 2024-11-27

**Authors:** Joseph Donovan, Marcel Wolbers, Nguyen Thuy Thuong Thuong, Dong Huu Khanh Trinh, Le Thanh Hoang Nhat, Guy E. Thwaites, Ronald B. Geskus

**Affiliations:** 1 Oxford University Clinical Research Unit, Centre for Tropical Medicine, Ho Chi Minh City, Vietnam; 2 Centre for Tropical Medicine and Global Health, Nuffield Department of Medicine, University of Oxford, Oxford, England, UK; 3 London School of Hygiene and Tropical Medicine, London, England, UK; 4 King's College London, London, England, UK

**Keywords:** tuberculous meningitis, corticosteroids, clinical trial, analysis plan, leukotriene A4 hydrolase (LTA4H), personalised medicine

## Abstract

Tuberculous meningitis (TBM) is the most severe form of tuberculosis. Corticosteroids are currently recommended as an adjunctive therapy in HIV-negative adults with TBM. However, benefit from corticosteroids in TBM may depend upon host *leukotriene A4 hydrolase* ( *LTA4H*) genotype and the corresponding inflammatory phenotypes. This article describes the planned analyses for the primary publication of the results of the LAST ACT clinical trial (NCT03100786): ‘Leukotriene A4 hydrolase Stratified Trial of Adjunctive Corticosteroids for HIV-negative adults with Tuberculous meningitis’. The primary hypothesis addressed by the trial is that *LTA4H* genotype, in particular CC or CT genotype, determines whether adjunctive dexamethasone benefits or harms adults with TBM. The trial was an *LTA4H* genotype stratified, parallel group, randomised, double blind, placebo-controlled multi-centre Phase III trial of dexamethasone given for 6–8 weeks in addition to standard anti-tuberculosis drugs. *LTA4H* genotype (CC, CT, TT) was determined in all participants prior to randomisation; only those with CC or CT genotype were randomised to dexamethasone or placebo. All TT genotype participants received dexamethasone because prior data indicated survival was increased by dexamethasone in this genotype. The primary endpoint was all-cause death or new neurological event over the first 12 months after randomisation. We took a hybrid trial-design approach which aims to prove non-inferiority of placebo first but also allows claiming superiority of placebo in case dexamethasone causes substantial harm. This statistical analysis plan expands upon and updates the analysis plan outlined in the published study protocol.

## Plain Language Summary

This article describes the planned analyses for the LAST ACT clinical trial, in which corticosteroids (or placebo) were given to HIV-negative adults with tuberculous meningitis stratified by their leukotriene A4 hydrolase (LTA4H) genotype. Tuberculous meningitis is the most severe form of tuberculosis. Dexamethasone (a corticosteroid) is currently standard of care for HIV-negative adults with tuberculous meningitis. However, patients who suffer from tuberculous meningitis can have one of three variations of part of the gene called LTA4H. These small variations in the gene's promotor region (a region that directs how the gene is expressed) affect the expression of the LTA4H gene. This results in an individual having one of three genotypes: TT, CT or CC, and therefore one of three corresponding inflammatory phenotypes of high inflammatory, intermediate inflammatory, and low inflammatory, respectively. Corticosteroids may not be equally effective for patients with tuberculous meningitis with each of these genotypes/phenotypes.

This trial’s primary objective is to determine whether placebo is not worse than dexamethasone in HIV-negative, CC or CT genotype adult patients, when added to the first 6–8 weeks of anti-tuberculosis treatment of tuberculous meningitis.

## Scope of document

This document outlines the planned analyses for the primary publication of the results of the LAST ACT clinical trial (‘a Leukotriene A4 hydrolase Stratified Trial of Adjunctive Corticosteroids for HIV-negative adults with Tuberculous meningitis’ [clinicaltrials.gov, NCT03100786, posted 04-04-2017, URL: https://clinicaltrials.gov/study/NCT03100786]). This statistical analysis plan expands upon and updates the analysis plan outlined in the published study protocol ^1^. Data will be reported following guidelines for “Reporting of Noninferiority and Equivalence Randomized Trials, Extension of the CONSORT 2010 Statement” ^2^.

## Background and rationale for study

Adjunctive dexamethasone is currently standard of care for HIV-negative adults with tuberculous meningitis (TBM) ^3, 4^. However, host *LTA4H* genotype may determine the effectiveness of adjunctive dexamethasone. *LTA4H* catalyses the final step in the synthesis of pro-inflammatory leukotriene B4 ^5, 6^. A single nucleotide polymorphism (rs17525495) in the promoter region of the *LTA4H* gene alters the gene’s expression, resulting in one of three genotypes: TT, CT or CC, and three corresponding inflammatory phenotypes of high inflammatory, intermediate inflammatory, and low inflammatory, respectively ^5^. In a retrospective study of adults with TBM in Vietnam, the use of adjunctive dexamethasone was associated with improved survival in high inflammatory TT genotype patients. However, the benefit of dexamethasone in CC and CT genotype patients was unclear.

## Trial objectives, design, and sample size

The trial’s primary objective was to determine whether placebo is not worse than dexamethasone in HIV-negative, CC or CT genotype adult patients when added to the first 6–8 weeks of anti-tuberculosis treatment of TBM. In a primary subgroup analysis, it addresses the question whether non-inferiority of placebo holds in the CC-genotype subgroup only. In principle, administration of dexamethasone in CC and CT genotypes would be discouraged if placebo could be shown to be non-inferior to dexamethasone. However, as the benefit of dexamethasone in the TT genotype is undisputed, and personalised administration of dexamethasone in HIV negative subjects would necessitate rapid genotype testing, some evidence of harm of dexamethasone in the CC/CT population (or the CC group alone) would be required to change clinical practice.

We therefore opted for a hybrid trial-design approach which aims to prove non-inferiority of placebo first but also allows claiming superiority of placebo in case dexamethasone proves to induce substantial harm. We allowed for early stopping of the trial for the CC and/or the CT group if either dexamethasone was shown to be beneficial or to be harmful. The Haybittle-Peto threshold was used, i.e., p<0.001.

Because the retrospective study of adults with TBM in Vietnam suggested that the harm of dexamethasone could be larger in the CC population, the trial was designed with two primary populations: the combined CC/CT population (designated 2% of the total one-sided type I error of 2.5%) and the CC population (designated the remaining type I error after exploiting the correlation between test statistics as described further in the analysis section below) ^7^.

We set the non-inferiority margin in favour of dexamethasone at a hazard ratio (HR) of 0.75 and assumed a true HR of 1.15 in the CC/CT population. Under these assumptions a total of 184 neurological events or deaths in the CC/CT population would be required to obtain 80% power in the combined CC/CT population at the one-sided 2% significance level. Assuming an absolute risk of a neurological event or death in the dexamethasone group by 12 months of 35%, a HR=1.15 corresponds to a risk of 31.2% of placebo, and the non-inferiority margin translates to an absolute risk increase of placebo of (at worst) +8.7%. Assuming an overall event risk of ≥32%, and an 11% sample size increase to compensate for loss-to-follow-up and reductions in power due to the allowance for stopping due to futility, around 640 HIV-negative subjects with CC or CT genotype will be randomised into the trial (720 in total, including TT genotype participants).

Apart from the primary analysis of the primary endpoint, analyses use a superiority design in which the null hypothesis assumes no difference between the treatment arms and no corrections for multiple testing are made.

## Structure and status of trial

The LAST ACT trial commenced recruitment on 12 ^th^ February 2018. By 9 ^th^ March 2023, the predefined sample size of 720 HIV negative adults with TBM, of either TT, CC or CT *LTA4H* genotype had been enrolled from two hospitals in Vietnam: The Hospital for Tropical Diseases (HTD) and Pham Ngoc Thach Hospital for Tuberculosis and Lung Disease (PNT), in Ho Chi Minh City. Detailed enrolment criteria for LAST ACT, including consent and ethical approvals, are described in the published trial protocol ^1^.

During trial design, it was estimated that in order to enrol and randomise 640 *LTA4H* CC and CT genotype participants, a total of 720 participants (of all *LTA4H* genotypes) would be recruited, based on prior population estimates of genotype frequencies. However, as trial completion approached, a higher-than-expected proportion of TT genotype participants had been recruited, which was set to result in a study population of 720 participants including less than 640 *LTA4H* CC and CT genotype participants. A decision was therefore taken, in conjunction with the Trial Steering Committee, to complete recruitment at a total study population of 720 participants rather than at 640 *LTA4H* CC and CT genotype participants. Given the lower than anticipated loss to follow up or study withdrawal in the trial, this decision was considered to not impact the trial.

**Figure 1.**
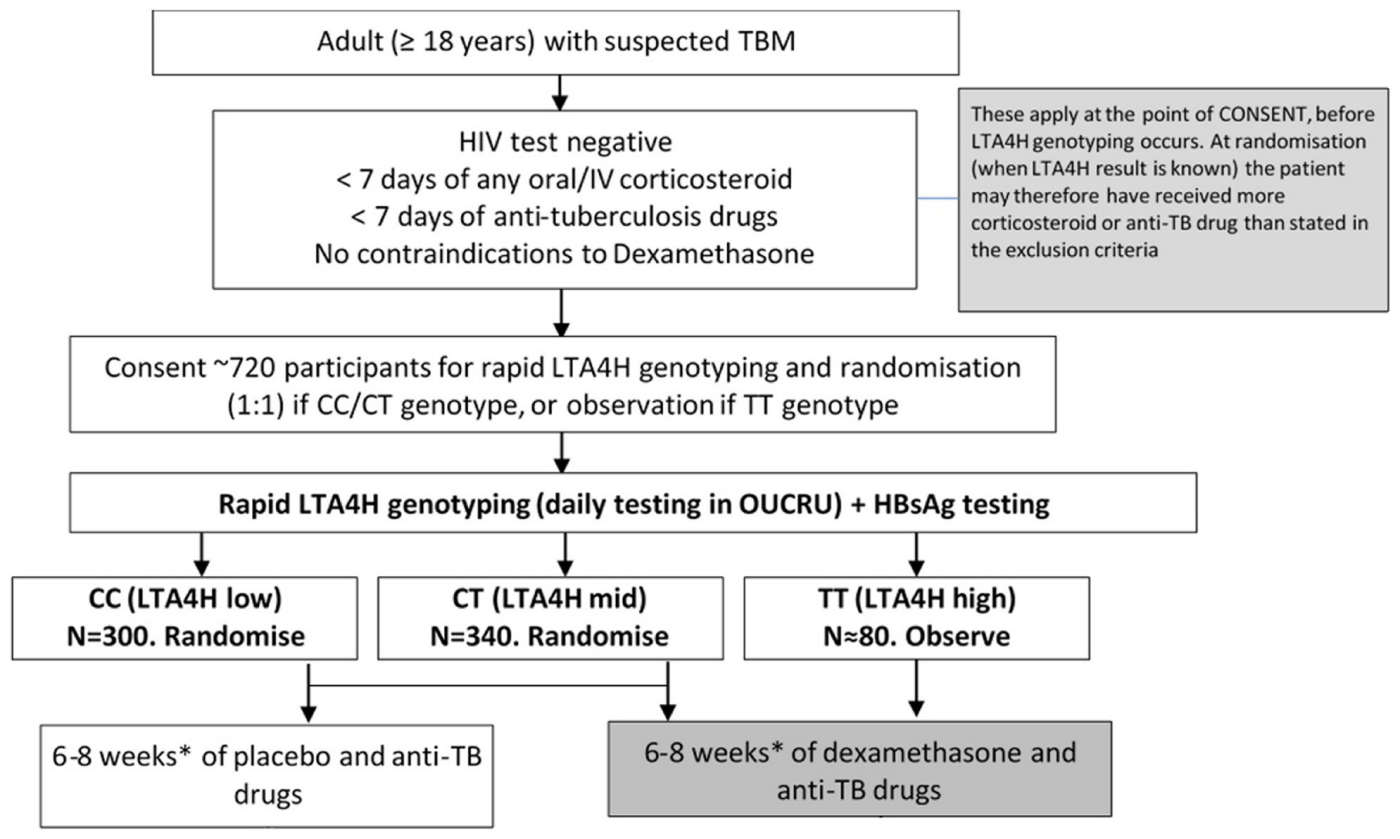
The trial schema. HIV=human immunodeficiency virus. *LTA4H=leukotriene A4 hydrolase*. N=number. OUCRU=Oxford University Clinical Research Unit. TBM=tuberculous meningitis.

Enrolled study participants of CC or CT *LTA4H* genotype were randomised to dexamethasone or placebo (a double-blinded allocation), with this intervention termed ‘study drug’. Randomisation was stratified by *LTA4H* genotype, the TBM Modified Medical Research Council (MRC) severity score, and by hospital. Participants with MRC grade 1 TBM received a 6-week tapering course of study drug, whereas participants with MRC grades 2 or 3 received an 8-week tapering course of study drug. Study drug regimens are shown in Table 1. Study participants of TT *LTA4H* genotype received dexamethasone following the same tapering course based on TBM MRC severity grade ( Figure 1). Enrolled participants and dexamethasone allocation in this non-inferiority trial are highly similar to those in the trial that established efficacy of the reference treatment ^4^.

**Table 1.** Study drug treatment regimen following randomisation.

	MRC Grade I Daily dexamethasone dose/route	MRC Grades II and III Daily dexamethasone dose/route
Week 1	0.3 mg/kg/24 hrs IV	0.4 mg/kg/24 hrs IV
Week 2	0.2 mg/kg/24 hrs IV	0.3 mg/kg/24 hrs IV
Week 3	0.1 mg/kg/24 hrs IV	0.2 mg/kg/24 hrs IV
Week 4	3mg/24 hrs oral	0.1 mg/kg/24 hrs IV
Week 5	2mg/24 hrs oral	4 mg/24 hrs oral
Week 6	1 mg/24 hrs oral	3 mg/24 hrs oral
Week 7	Stop	2 mg/24 hrs oral
Week 8		1 mg/24 hrs oral

hrs=hours. IV=intravenous. kg=kilograms. mg=miligrams. MRC=Medical Research Council

Participants underwent clinical assessments at baseline (day of randomisation), at days 3, 7, 10, 14, 21, and 30, and monthly until month 12. Baseline assessment included blood tests, chest X-ray, lumbar puncture, and brain imaging. LAST ACT sub-studies are described in the study protocol ^1^, and match those of the paired ACT HIV clinical trial (NCT03092817) ^8, 9^.

## Trial endpoints

### Primary endpoint

The primary endpoint of the LAST ACT trial was all-cause mortality or new neurological event, whichever is earlier, over the first 12 months after randomisation. A new neurological event was defined as follows: new cerebellar symptoms; hemiplegia, paraplegia, tetraplegia or monoplegia; seizures; cerebral herniation; cranial nerve palsy; or a fall in Glasgow coma score by ≥2 points for ≥2 days (from the highest previously recorded GCS, including baseline).

### Secondary endpoints

The secondary endpoints of the LAST ACT trial are as follows:

1. Death over the first 12 months after randomisation

2. First new neurological event over the first 12 months after randomisation

3. Use of open-label corticosteroid treatment for any reason over the first 12 months after randomisation

4. Neurological disability (defined as modified Rankin score ≥3) at 12 months from randomisation

5. All modified Rankin scores as an ordinal scale at 12 months from randomisation

6. Measurements of blood and cerebrospinal fluid inflammation

7. Severe (grade 3&4) and serious adverse events until 12 months from randomisation

## Individuals included in analyses

### Intention-to-treat

An intention-to-treat (ITT) analysis will be performed for the primary and secondary endpoints. This analysis will include all randomised participants of CC or CT genotype (i.e., excluding TT genotype participants). Participants will remain included in this analysis even if no study drug was received after randomisation. Participants who were enrolled (genotyped) but not randomised will not be included in the ITT or per protocol analysis. For the assessment of non-inferiority in the second primary population, the CC genotype subpopulation, the subset of ITT subjects with a CC genotype will be analysed.

### Per protocol analysis

The per protocol (PP) analysis includes all randomised participants with the exception of those subsequently found to have not met all inclusion criteria, or to have met any exclusion criteria at the time of enrolment; participants with a final diagnosis other than TBM subsequent to randomisation (confirmed by microbiology, serology, or histopathology). We also exclude participants who received less than 7 days of administration of the randomised study drug for reasons other than death, or less than 30 days of anti-tuberculosis drugs for any reason other than death. We assume the reason for this incomplete drug administration not to be related to progression to the end points of interest, and therefore there is no risk of collider selection bias. . A PP analysis will be performed for all primary and secondary endpoints.

## Statistical software

Data will be analysed using the program R ^10^, using the most up to date version available at the time of final analysis.

## Baseline characteristics

Definition: The following baseline characteristics will be summarised by treatment arm (excluding TT group) for ITT and PP analyses: age, sex, site, *LTA4H* genotype (CC/CT), diagnostic category (definite, probable, possible, or not TBM by Marais criteria ^11^), history of previous tuberculosis treatment, chest X-ray findings (no TB/miliary TB/pulmonary TB), enrolment MRC TBM grade, enrolment Glasgow coma score (GCS), weight, duration of symptoms of TBM, cranial nerve palsy, hemiplegia, paraplegia, tetraplegia, urinary retention, history of diabetes, HbA1c, hepatitis B sAg positivity, hepatitis C Ab positivity, alanine aminotransferase (ALT), bilirubin, full blood count (haemoglobin, white cell count, platelets), plasma sodium, routine cerebrospinal fluid (CSF) parameters (opening pressure, total leucocytes, total neutrophils, total lymphocytes, protein, CSF:blood glucose ratio, Ziehl-Neelsen stain, Gene Xpert MTB/RIF, Gene Xpert MTB/RIF Ultra, mycobacterial culture), duration of anti-tuberculosis chemotherapy before enrolment, enrolment anti-TB regimen, anti-TB drug susceptibility results in culture-confirmed sub-group (multi-drug resistant [MDR] TB or rifampicin mono-resistant TB, isoniazid resistant non-MDR, or no or other resistance). These will be described in a baseline variable table ( Table 2). We will summarize the same variables by genotype, not stratified by treatment arm.

**Table 2.** Baseline characteristics template table.

Characteristic	Dexamethasone (N=…)		Placebo (N=…)	
	N	Summary statistic	N	Summary statistic
Age (years)	XX	XX (XX,XX)	XX	XX (XX,XX)
Sex – male	XX	XX (XX%)	XX	XX (XX%)
Site - HTD - PNT	XX XX	XX (XX%) XX (XX%)	XX XX	XX (XX%) XX (XX%)
*Leukotriene A4 hydrolase* - TT - CT - CC	XX XX XX	XX (XX%) XX (XX%) XX (XX%)	XX XX	XX (XX%) XX (XX%)
Diagnostic category * - Definite TBM - Probable TBM - Possible TBM - Not TBM	XX XX XX XX	XX (XX%) XX (XX%) XX (XX%) XX (XX%)	XX XX XX XX	XX (XX%) XX (XX%) XX (XX%) XX (XX%)
Previous tuberculosis treatment	XX	XX (XX%)	XX	XX (XX%)
Chest X-ray findings - No tuberculosis - Miliary tuberculosis - Pulmonary tuberculosis	XX XX XX	XX (XX%) XX (XX%) XX (XX%)	XX XX XX	XX (XX%) XX (XX%) XX (XX%)
Modified MRC grade - Grade I - Grade II - Grade III	XX XX XX	XX (XX%) XX (XX%) XX (XX%)	XX XX XX	XX (XX%) XX (XX%) XX (XX%)
Glasgow coma score	XX	XX (XX,XX)	XX	XX (XX,XX)
Weight (kg)	XX	XX (XX,XX)	XX	XX (XX,XX)
Duration of symptoms (days)	XX	XX (XX,XX)	XX	XX (XX,XX)
Neurological signs - Cranial nerve palsy - Hemiplegia - Paraplegia/tetraplegia - Urinary retention	XX XX XX XX	XX (XX%) XX (XX%) XX (XX%) XX (XX%)	XX XX XX XX	XX (XX%) XX (XX%) XX (XX%) XX (XX%)
History of diabetes	XX	XX (XX%)	XX	XX (XX%)
HbA1c (%)	XX	XX (XX,XX)	XX	XX (XX,XX)
Hepatitis B sAg positivity,	XX	XX (XX%)	XX	XX (XX%)
Hepatitis C Ab positivity	XX	XX (XX%)	XX	XX (XX%)
Alanine aminotransferase (ALT) (IU/L)	XX	XX (XX,XX)	XX	XX (XX,XX)
Bilirubin (µmol/L)	XX	XX (XX,XX)	XX	XX (XX,XX)
Full blood count - Haemoglobin (g/dL) - White cell count (x10 ^3^/ µL) - Platelets (x10 ^3^/ µL)	XX XX XX	XX (XX,XX) XX (XX,XX) XX (XX,XX)	XX XX XX	XX (XX,XX) XX (XX,XX) XX (XX,XX)
Plasma sodium (mmol/L)	XX	XX (XX,XX)	XX	XX (XX,XX)
CSF parameters - Opening pressure (cmH _2_0) - Total leucocytes (cells/mm ^3^) - Total neutrophils (cells/mm ^3^) - Total lymphocytes (cells/mm ^3^) - Protein (g/L) - CSF:blood glucose	XX XX XX XX XX XX	XX (XX,XX) XX (XX,XX) XX (XX,XX) XX (XX,XX) XX (XX,XX) XX (XX,XX)	XX XX XX XX XX XX	XX (XX,XX) XX (XX,XX) XX (XX,XX) XX (XX,XX) XX (XX,XX) XX (XX,XX)
CSF microbiological tests - Positive ZN stain - Positive GeneXpert MTB/RIF - Positive GeneXpert MTB/RIF Ultra - Positive mycobacterial culture	XX XX XX XX	XX (XX%) XX (XX%) XX (XX%) XX (XX%)	XX XX XX XX	XX (XX%) XX (XX%) XX (XX%) XX (XX%)
Duration of anti-tuberculosis chemotherapy before enrolment (days)	XX	XX (XX,XX)	XX	XX (XX,XX)
Enrolment anti-tuberculosis chemotherapy regimen - Rifampicin - Isoniazid - Pyrazinamide - Ethambutol - Streptomycin	XX XX XX XX XX	XX (XX%) XX (XX%) XX (XX%) XX (XX%) XX (XX%)	XX XX XX XX XX	XX (XX%) XX (XX%) XX (XX%) XX (XX%) XX (XX%)
Anti-tuberculosis drug resistance ^#^ - Multi-drug resistant or rifampicin mono-resistant - Isoniazid resistant non-MDR - No or other resistance	XX XX XX	XX (XX%) XX (XX%) XX (XX%)	XX XX XX	XX (XX%) XX (XX%) XX (XX%)

* Participants will only be categorised as ‘not TBM’ if they have a confirmed alternative diagnosis (alternative to TBM) or if they fully recovered without any anti-TB drugs. ^#^Results given for sub-group with positive mycobacterial culture on baseline CSF. N = number of patients included in that statistic. Summary statistic = the median (1 ^st^ and 3 ^rd^ quartile) value for numeric data, and the number and frequency (%) of patients with the characteristic for categorical data. Definite TBM = positive acid fast bacilli (AFB) on CSF Ziehl Neelsen stain, or positive CSF TB GeneXpert test, OR positive CSF TB culture. Probable or possible TBM defined following uniform case defintion, ^10^ with the modification that participants with score<6, who do not meet the criteria for ‘not tuberculous meningitis’, will be classifed as ‘possible TBM’. Confirmed not-TBM = microbiologically confirmed other brain infection. Confirmed additional brain infection includes positive CSF India Ink stain, or CSF cryptococcal antigen, or positive blood cryptococcal antigen, or positive CSF bacterial Gram stain, or positive CSF bacterial culture, or positive CSF viral or helminth PCR test. HTD=Hospital for Tropical Diseases. MDR=multi-drug resistant. MRC=Medical Research Council. PNT=Pham Ngoc Thach Hospital for Tuberculosis and Lung Disease. TBM=tuberculous meningitis. ZN=Ziehl Neelsen.

Analysis: Baseline characteristics will be summarised as median (lower and upper quartiles) for continuous data and frequency (percentage) for categorical data. The amount of missing data for each baseline characteristic will also be displayed.

## Use of the uniform case definition diagnostic score

The published TBM diagnostic score ^11^ will be used and subjects will be categorised as ‘definite’, ‘probable’, ‘possible’, or ‘not TBM’. Participants will only be categorised as ‘not TBM’ if they have a confirmed alternative diagnosis (alternative to TBM) or if they fully recovered without any anti-TB drugs. Participants with a TBM diagnostic score <6, who do not meet the criteria for ‘not tuberculous meningitis’, will be classified as ‘possible TBM’. For the subgroup analyses we will perform multiple imputation of a variable in case of >5% missing values, and it is assumed that data are missing at random. Otherwise, a complete case analysis will be performed.

### Primary endpoint

The primary analysis is a Cox proportional hazards regression model with the primary endpoint as the outcome, treatment as the only covariate, and with *LTA4H* genotype (CC or CT) and TBM MRC severity grade at enrolment (I, II, or III) as stratum variables. We will also perform the analysis in the CC subgroup only (with TBM MRC severity grade at enrolment as stratum variable). Non-inferiority of placebo in the CC/CT population or the CC genotype subgroup will be established if the corresponding test rejects the null hypothesis that dexamethasone decreases the hazard of the primary endpoint by 25% or more. To protect the one-sided overall familywise error rate of 2.5% for the analysis of the primary endpoint across the two co-primary populations (the full CC/CT population and the CC population), we will assign a multiplicity-corrected one-sided significance level of 2% to the full population and of 0.84% to the CC population exploiting the correlation between test statistics on the two populations using the Spiessens and Debois method ^7^. The exact significance level for the CC subgroup test will be updated based on the actual number of events in CC and CT in the final analysis dataset.

P-values for non-inferiority will not be calculated. Rather, the null hypothesis of the non-inferiority comparison will be tested indirectly via the calculation of confidence intervals for the treatment effect with confidence levels corresponding to the multiplicity-adjusted significance levels (e.g., a two-sided 96% confidence interval corresponding to a one-sided significance level of 2%). If this confidence interval is fully located to the right of the HR of 0.75, then the null hypothesis of the non-inferiority test can be rejected and non-inferiority is established. We will also quantify confidence intervals while protection the familywise error rate at 0.5% and 5%, assigning a one-sided significance level of 0.4% and 4% respectively to the test for CC&CT combined. Superiority of placebo will additionally be established, if the null hypothesis that dexamethasone does not affect the hazard of the primary endpoint can be rejected against the one-sided alternative that dexamethasone causes harm. This is based on the same significance levels and confidence intervals; p-values will be given as well.

Due to the issue of non-collapsibility, correction for strong predictors of the outcome is not guaranteed to increase power in a non-inferiority design ^12^. Therefore, the same analyses will be performed using a Cox model without MRC grade and *LTA4H* included, but the analysis with strata will be considered the primary one.

The proportional hazards assumption will be formally tested based on scaled Schoenfeld residuals and visually assessed by a plot of the scaled Schoenfeld residuals versus time transformed via the Kaplan-Meier estimate.

All further analyses use a superiority design, and no corrections are made for multiple testing. The distribution of the primary endpoint will also be visualised using Kaplan-Meier plots and their difference by treatment arm, with stratification by genotype (CC, CT, and CC/CT combined), and explicit survival estimates at 3, 6, 9, and 12 months of follow-up will be calculated. A formal comparison between the two arms of the restricted mean time lost (RMTL) until 12 months will be performed. We use a regression model with a linear link; for estimation we use the approach based on pseudo-values. We perform the analysis for both the CC/CT groups combined (correcting for genotype and MRC grade) as well as for the CC group alone (correcting for MRC grade).

The homogeneity of the treatment effect on the primary endpoint across subgroups will be assessed by subgroup analyses, using a Cox proportional hazards model. We will fit separate models per subgroup and we will fit a model in which we test for interaction between treatment and subgroup. We will consider the following grouping variables: *LTA4H* genotype, TBM MRC severity grade at enrolment (I, II, or III), TBM diagnosis (definite, probable, possible), and drug resistance pattern (MDR-TB or rifampicin mono-resistance, isoniazid resistant non-MDR, no or other resistance). No stratification variables (genotype and MRC grade) are included in these analyses. See Table 3 and Table 4 for the presentation of the results. We will additionally use a model for the RMTL (with linear link function), and use a similar presentation of the results as in Table 3 and Table 4.

**Table 3.** Primary non-inferiority analysis for null hypothesis HR ≤ 0.75; All-cause mortality or new neurological event until 12 months after randomisation.

	All-cause mortality or new neurological event		Hazard ratio (CI) *
	Dexamethasone	Placebo	
All patients of CT or CC genotype	XX/XX	XX/XX	X.XX (X.XX, X.XX)
All patients of CC genotype	XX/XX	XX/XX	X.XX (X.XX-X.XX)

* CI=confidence interval. Significance level will be based on the Spiessens and Debois method XX/XX denotes the number of participants experiencing the event divided by the total number of participants in the treatment arm

**Table 4.** Primary endpoint; superiority subgroup analyses.

	All-cause mortality or new neurological event		Hazard ratio (95% CI)	p-value for superiority	p-value for heterogeneity *
	Dexamethasone	Placebo			
*LTA4H* genotype - CT - CC	XX/XX XX/XX	XX/XX XX/XX	X.XX (X.XX-X.XX) X.XX (X.XX-X.XX)	X.XX X.XX	X.XX
Modified MRC grade - Grade I - Grade II - Grade III	XX/XX XX/XX XX/XX	XX/XX XX/XX XX/XX	X.XX (X.XX-X.XX) X.XX (X.XX-X.XX) X.XX (X.XX-X.XX)	X.XX X.XX X.XX	X.XX
TBM diagnosis - Definite - Probable - Possible	XX/XX XX/XX XX/XX	XX/XX XX/XX XX/XX	X.XX (X.XX-X.XX) X.XX (X.XX-X.XX) X.XX (X.XX-X.XX)	X.XX X.XX X.XX	X.XX
Drug resistance ^#^ - Rifampicin resistance - Isoniazid mono-resistance- - Susceptible to rifampicin and isoniazid	XX/XX XX/XX XX/XX	XX/XX XX/XX XX/XX	X.XX (X.XX-X.XX) .XX (X.XX-X.XX) X.XX (X.XX-X.XX)	X.XX X.XX X.XX	X.XX

* Heterogeneity is tested with a Cox regression model that includes an interaction between treatment effect and subgroup. ^# ^Rifampicin resistance: Tuberculosis resistant to rifampicin, with or without any other drug; Isoniazid mono-resistance: Tuberculosis resistant to isoniazid, either alone or in combination with any drug except rifampicin; Susceptible to rifampicin and isoniazid: Tuberculosis susceptible to rifampicin and isoniazid, with or without resistance to other drugs. XX/XX denotes the number of participants experiencing the event divided by the total number of participants in the treatment arm CI=confidence interval. *LTA4H*= *leukotriene A4 hydrolase*. MRC=Medical Research Council.

Survivors known to be alive at 12 months will be censored at that time-point and subjects who withdrew or were lost to follow-up before 12 months will be censored at the date they were last known to be alive. Subjects who withdrew or were lost to follow-up before 12 months are estimated to be less than 5% of enrolled participants. 

### Secondary endpoints

Secondary outcomes will be compared in all those randomised (as with the primary outcome analysis, TT genotype participants will be excluded from the analyses). We will perform the analyses for both the CC/CT groups combined (correcting for *LTA4H* genotype and MRC grade) as well as for the CC group alone (correcting for MRC grade), with the exception of adverse events which will be described and compared by genotype and the blood and CSF inflammatory measurements (outcome 6), the analysis of which are described below.

Outcomes 1–3 below are time-to-event outcomes, and we use similar analyses as for the primary outcome: we compare RMTL at 12 months as well as the HR based on a Cox proportional hazards model (results are interpreted as relative cause-specific hazards in the presence of competing risks, which do not directly relate to the cause-specific cumulative incidence as is quantified by the RMTL). For each, we will also compute and plot the Kaplan-Meier estimates (outcome 1) or the Aalen-Johansen estimates with death as competing risk and plot them in appropriate format (overlaid or alternate, outcomes 2 and 3), with stratification by genotype, and report the values of the estimates at 3, 6 and 12 months. 

We perform subgroup analyses for *LTA4H* genotype, TBM MRC severity grade and final diagnosis (uniform case definition for TBM ^11^) for the primary outcome, and outcomes 1-3 below. We follow the same procedure as for the primary outcome: we will fit separate models per subgroup (without correction for genotype nor MRC grade) and we will fit a model in which we test for interaction by subgroup.

All results are presented via tables (with p-values and 95% confidence intervals per subgroup) as well as via forest plots (with 95% confidence intervals per subgroup).

The secondary outcomes are:

***1. Death over the first 12 months after randomisation***

Overall survival will be analysed. See Table 5 for the results from the Cox model. A similar presentation is used for the results based on the restricted mean time lost.

**Table 5.** Secondary endpoint; Death over the first 12 months after randomisation.

	Death		Hazard ratio (95% CI)	p-value	p-value for heterogeneity *
	Dexamethasone	Placebo			
All patients of CT or CC genotype	XX/XX	XX/XX	X.XX (X.XX, X.XX)	X.XX	
*LTA4H* genotype - TT - CT - CC	XX/XX XX/XX XX/XX	XX/XX XX/XX	X.XX (X.XX-X.XX) X.XX (X.XX-X.XX)	X.XX X.XX	X.XX
Modified MRC grade - Grade I - Grade II - Grade III	XX/XX XX/XX XX/XX	XX/XX XX/XX XX/XX	X.XX (X.XX-X.XX) X.XX (X.XX-X.XX) X.XX (X.XX-X.XX)	X.XX X.XX X.XX	X.XX
TBM diagnosis - Definite - Probable - Possible	XX/XX XX/XX XX/XX	XX/XX XX/XX XX/XX	X.XX (X.XX-X.XX) X.XX (X.XX-X.XX) X.XX (X.XX-X.XX)	X.XX X.XX X.XX	X.XX

* Heterogeneity was tested with a Cox regression model that included an interaction between treatment effect and subgroup. CI=confidence interval. *LTA4H*= *leukotriene A4 hydrolase*. MRC=Medical Research Council.

***2. First new neurological event over the first 12 months after randomisation***

New neurological events will be analysed. This analysis will include a clinical description of neurological events, and their time of onset. See Table 6 for the results from the Cox model. A similar presentation is used for the results based on the restricted mean time lost.

**Table 6.** First new neurological event over the first 12 months after randomisation.

	First new neurological event		Hazard ratio (95% CI)	p-value	p-value for heterogeneity *
	Dexamethasone	Placebo			
All patients of CT or CC genotype	XX/XX	XX/XX	X.XX (X.XX, X.XX)	X.XX	
*LTA4H* genotype - TT - CT - CC	XX/XX XX/XX XX/XX	XX/XX XX/XX	X.XX (X.XX-X.XX) X.XX (X.XX-X.XX)	X.XX X.XX	X.XX
Modified MRC grade - Grade I - Grade II - Grade III	XX/XX XX/XX XX/XX	XX/XX XX/XX XX/XX	X.XX (X.XX-X.XX) X.XX (X.XX-X.XX) X.XX (X.XX-X.XX)	X.XX X.XX X.XX	X.XX
TBM diagnosis - Definite - Probable - Possible	XX/XX XX/XX XX/XX	XX/XX XX/XX XX/XX	X.XX (X.XX-X.XX) X.XX (X.XX-X.XX) X.XX (X.XX-X.XX)	X.XX X.XX X.XX	X.XX

* Heterogeneity was tested with a Cox regression model that included an interaction between treatment effect and subgroup. CI=confidence interval. *LTA4H*= *leukotriene A4 hydrolase*. MRC=Medical Research Council.

***3. Use of open-label corticosteroid treatment for any reason over the first 12 months after randomisation***

Time to start of open-label corticosteroid treatment will be analysed. The number and proportion of subjects requiring ‘rescue’ corticosteroids will be summarised by treatment arm ( Table 7). This analysis will include a clinical description of events that required open-label corticosteroids, and the time of use of open-label corticosteroids. See Table 8 for the results from the Cox model. A similar presentation is used for the results based on the restricted mean time lost. 

**Table 7.** Use of open-label corticosteroid treatment for any reason over the first 12 months after randomisation.

Reason for open label corticosteroids	Dexamethasone N=…			Placebo N=…		
	Number of patients receiving open label corticosteroids	Number of episodes of open label corticosteroid use	Days from randomisation until use of open-label corticosteroids (median [IQR])	Number of patients receiving open label corticosteroids	Number of episodes of open label corticosteroid use	Days from randomisation until use of open-label corticosteroids (median [IQR])
All patients	XX/XX (XX%)	XX	XX (XX, XX)	XX/XX (XX%)	XX	XX (XX, XX)
Reason for use - Individual reasons	XX/XX (XX%)	XX	XX (XX, XX)	XX/XX (XX%)	XX	XX (XX, XX)

XX/XX denotes the number of participants experiencing the event divided by the total number of participants in the treatment arm. IQR=interquartile range.

**Table 8.** Use of open-label corticosteroid by sub-group.

	Use of open-label corticosteroid		Hazard ratio (95% CI)	p-value	p-value for heterogeneity *
	Dexamethasone	Placebo			
All patients of CT or CC genotype	XX/XX	XX/XX	X.XX (X.XX, X.XX)	X.XX	
*LTA4H* genotype - TT - CT - CC	XX/XX XX/XX XX/XX	XX/XX XX/XX	X.XX (X.XX-X.XX) X.XX (X.XX-X.XX)	X.XX X.XX	X.XX
Modified MRC grade - Grade I - Grade II - Grade III	XX/XX XX/XX XX/XX	XX/XX XX/XX XX/XX	X.XX (X.XX-X.XX) X.XX (X.XX-X.XX) X.XX (X.XX-X.XX)	X.XX X.XX X.XX	X.XX
TBM diagnosis - Definite - Probable - Possible	XX/XX XX/XX XX/XX	XX/XX XX/XX XX/XX	X.XX (X.XX-X.XX) X.XX (X.XX-X.XX) X.XX (X.XX-X.XX)	X.XX X.XX X.XX	X.XX

* Heterogeneity was tested with a Cox regression model that included an interaction between treatment effect and subgroup. XX/XX denotes the number of participants experiencing the event divided by the total number of participants in the treatment arm CI=confidence interval. *LTA4H*= *leukotriene A4 hydrolase*. MRC=Medical Research Council.

***4. Neurological disability at 12 months from randomisation***

Neurological disability, which denotes an inability to live independently of others, has been defined in earlier TBM trials ^4^ as modified Rankin score ≥3. We will therefore use this cut-off to dichotomise outcomes at 12 months. Individuals who died before 12 months will be treated as having a score of 6 (‘dead’) ( Table 9). Individuals who withdrew or were lost to follow-up before 12 months will be excluded. For this 12-month assessment an acceptable range of -10 days/+1 month will be applied. Twelve-months timing is based on days from randomisation (i.e., ‘day 0’ is labelled as the first day study drug is received, with study drug received immediately after randomisation), or based on days from starting open label corticosteroid in the non-randomised TT genotype group. Neurological disability will be compared between the two arms via a logistic regression analysis.

**Table 9.** The Modified Rankin Scale.

Score	Description
0	No symptoms
1	Minor symptoms not interfering with lifestyle
2	Symptoms that lead to some restriction in lifestyle, but do not interfere with the patient’s ability to look after themselves
3	Symptoms that restrict lifestyle and prevent totally independent living
4	Symptoms that clearly prevent independent living, although the patient does not need constant care and attention.
5	Totally dependent, requiring constant help day and night.
6	Death

***5. All modified Rankin scores as an ordinal scale at 12 months from randomisation***

All modified Rankin scores, as assessed by the ordinal modified Rankin scale at 12 months will be compared between the two arms with a proportional odds regression model ( Table 10). The result will be summarised as a cumulative odds ratio. Individuals who withdrew or were lost to follow-up before 12 months are excluded.

**Table 10.** Neurological disability at 12 months from randomisation.

12-month Modified Rankin Score	Dexamethasone (N=…)		Placebo (N=…)	
	n	Summary statistic	n	Summary statistic
- 0		XX/n (XX%)		XX/n (XX%)
- 1		XX/n (XX%)		XX/n (XX%)
- 2		XX/n (XX%)		XX/n (XX%)
- 3		XX/n (XX%)		XX/n (XX%)
- 4		XX/n (XX%)		XX/n (XX%)
- 5		XX/n (XX%)		XX/n (XX%)

n = number of patients included in that statistic, with individuals who died or were lost to follow-up before 12 months excluded.

***6. Measurements of blood and cerebrospinal fluid inflammation***

The whole blood transcriptomic data were measured from the first 207 patients enrolled consecutively into the trial with three time-points following randomisation (day 0, day 14, day 60). Inflammatory CSF proteins were measured by Olink (Explore 384 inflammation proteomics, including 10 reported cytokines [TNF-a, IL-1b, IL-2, IL-6, IL-12p70, IFN-y, IL-4, IL-5, IL-10, IL-13]) ^13^ from all recruited patients with two time-points (day 0, day 30).

Analyses will focus on five targeted pathways known to be important mediators of TB or TBM pathogenesis: TNF signalling, interferons, cytokine signalling, neutrophil mediated immunity, and eicosanoids and 10 reported cytokines. In particular, for each targeted pathway, we will perform single sample gene set enrichment analysis ^14^ ( *ssGSEA*) and pathway activity (zscore ^15^) to evaluate the enrichment score of the pathway at each time point for both whole blood transcriptomics and CSF proteomics, respectively, for each patient.

We will visualise the individual enrichment score for each targeted pathway by follow-up time in a scatter plot, separated by colour for treatment arms and genotypes. We will use an appropriate transformation, such as log transformation, for enrichment score to obtain a fairly symmetric variation and a linear trend by follow-up time if necessary.

We will assess the effects of all three *LTA4H* genotypes on baseline transcriptomic and proteomic signatures. We will then assess the effect of treatment and their interaction with the *LTA4H* genotypes on the inflammatory response during follow-up on both transcriptomic and proteomic signatures. In particular, we will perform two analyses:

1) Estimate the effect of treatment arm and the effect modification by the two genotypes of *LTA4H* (CT vs. CC) on the targeted pathways, quantified via the difference in slope.

2) Estimate the difference of slope of signatures over time between genotypes (CT, CC vs. TT) of targeted pathways within the dexamethasone arm.

For the first analysis, the difference in slope since baseline between treatment arm overall and for subgroups (CC & CT) will be presented. The slope of enrichment score of each targeted pathway will be modelled based on a joint model consisting of a survival model and a linear mixed effects model with longitudinal enrichment scores as the outcome. In the linear mixed effect model, we will model the treatment arm (Rx), genotype ( *LTA4H*) as well as MRC severity grade (MRC), and the follow-up time (t) and their interactions as fixed covariates with specific R command (with piecewise linear trend for blood transcriptomics ~ ( *LTA4H* + MRC) × (pmin(t,14)+pmax(t,14)+ Rx+Rx: (pmin(t,14)+pmax(t,14)); linear trend for CSF proteomics ~ ( *LTA4H*+MRC)*t+Rx+Rx:t). We will use random patient-specific intercepts and slopes. The survival model considers mortality up to three months and includes treatment and the fitted value of enrichment scores as covariate. The model will be implemented in a Bayesian framework using R package JMBayes ^16^. This joint model would help to adjust for missing data from informative dropout due to early death within the first 60 days for transcriptomics and the first 30 days for proteomics following randomisation. The second analysis will be based on the similar model for the dexamethasone arm only. Hence, the linear mixed effect model consists of genotype (TT, CT, vs. CC), follow-up time and their interactions as fixed covariate, as well as MRC severity grade as main effect. We will also perform a sensitivity analysis to see whether the enrichment score at baseline differs among the three *LTA4H* genotypes, using a linear regression model with genotype and MRC as covariates. The *ssGESA* and zscore will be implemented in R package “GSVA” ^17^. All or some of the data from the above analysis may be published with the primary analysis, or published in a separate manuscript.

***7. Severe (grade 3&4) and serious adverse events until 12 months from randomisation***

Serious adverse events (SAE) are defined in the study protocol ^1^. SAE will be sub-grouped into categories. SAE will be grouped and graded as per Common Terminology Criteria for Adverse Events (CTCAE) ^13^. The number of patients with any serious adverse event will be summarised and compared between the two treatment arms based on the chi-squared test, or Fisher’s exact test in case the expected count under the null hypothesis in at least one of the cells is smaller than one ^14^. Specific adverse events will be summarised, but not formally compared. The total number of serious adverse event episodes per patient will also be summarised and informally compared based on a quasi-Poisson regression model with treatment as the only covariate and total follow-up time as offset.

The following subgroups of adverse events will also be separately summarised: clinical grade 3&4 adverse events; serious adverse events possibly, probably, or definitely related to the study drug; adverse events leading to TB treatment. Grade 3&4 laboratory abnormalities will be summarised in the same way as clinical adverse events. Adverse events will be shown as per Table 11– Table 16.

**Table 11.** Summary of serious adverse events.

	Dexamethasone		Placebo		P value
Characteristic	(N=…)		(N=…)		
	N.pt	N.ae	N.pt	N.ae	
CC genotype: any serious adverse event	XX (XX%)	XX	XX (XX%)	XX	XX
CC genotype: serious adverse events listed by type	XX (XX%)	XX	XX (XX%)	XX	
CT genotype: any serious adverse event	XX (XX%)	XX	XX (XX%)	XX	XX
CT genotype: serious adverse events listed by type	XX (XX%)	XX	XX (XX%)	XX	
TT genotype: any serious adverse event	XX (XX%)	XX			
TT genotype: serious adverse events listed by type	XX (XX%)	XX			

Serious adverse events will be formally compared between dexamethasone and placebo groups, for the each of the CC and CT genotype groups. All TT genotype participants received dexamethasone. N.pt = the number of patients with at least one serious adverse event (% of all patients receiving the same intervention). N.ae = the total number of episodes of that particular serious adverse event

**Table 12.** Summary of clinical grade 3&4 adverse events.

	Dexamethasone		Placebo	
Characteristic	(N=…)		(N=…)	
	N.pt	N.ae	N.pt	N.ae
CC genotype: any grade 3 or 4 adverse event	XX (XX%)	XX	XX (XX%)	XX
CC genotype: any grade 3 adverse event	XX (XX%)	XX	XX (XX%)	XX
CC genotype: any grade 4 adverse event	XX (XX%)	XX	XX (XX%)	XX
CC genotype: grade 3 or 4 adverse events listed by type	XX (XX%)	XX	XX (XX%)	XX
CT genotype: any grade 3 or 4 adverse event	XX (XX%)	XX	XX (XX%)	XX
CT genotype: any grade 3 adverse event	XX (XX%)	XX	XX (XX%)	XX
CT genotype: any grade 4 adverse event	XX (XX%)	XX	XX (XX%)	XX
CT genotype: grade 3 or 4 adverse events listed by type	XX (XX%)	XX	XX (XX%)	XX
TT genotype: any grade 3 or 4 adverse event	XX (XX%)	XX		
TT genotype: any grade 3 adverse event	XX (XX%)	XX		
TT genotype: any grade 4 adverse event	XX (XX%)	XX		
TT genotype: grade 3 or 4 adverse events listed by type	XX (XX%)	XX		

All TT genotype participants received dexamethasone. N.pt = the number of patients with at least one event (% of all patients receiving the same intervention). N.ae = the total number of episodes of that particular event

**Table 13.** Summary of serious adverse events, shown by reasons for which they were considered serious, not shown by study arm.

	Causes death (N=XX)	Life threatening event * (N=XX)	Hospitalisation of prolongation of hospitalisation (N=XX)	Persistent or significant disability/ incapacity ** (N=XX)	Congenital anomaly/birth defect (N=XX)	Important medical event which may jeopardise the patient and/or require intervention (N=XX)
	N Summary statistic	n Summary statistic	N Summary statistic	n Summary statistic	n Summary statistic	n Summary Statistic
Name of event	XX (XX%)	XX (XX%)	XX (XX%)	XX (XX%)	XX (XX%)	XX (XX%)

* Subjects were at immediate risk of death at the time of the event; it does not refer to an event which hypothetically might have caused death if it were more severe. ** A substantial disruption of a person's ability to conduct normal life functions. N is the number of all patients; n is the number of patients with a non-missing value. Summary statistic is absolute count (%) for categorical variable(s).

**Table 14.** Summary of serious adverse events possibly, probably, or definitely related to the study drug.

	Dexamethasone		Placebo	
Characteristic	(N=…)		(N=…)	
	N.pt	N.ae	N.pt	N.ae
CC genotype: Any serious adverse event possibly, probably, or definitely related to the study drug	XX (XX%)	XX	XX (XX%)	XX
CC genotype: Serious adverse events, possibly, probably, or definitely related to the study drug, listed by type	XX (XX%)	XX	XX (XX%)	XX
CT genotype: Any serious adverse event possibly, probably, or definitely related to the study drug	XX (XX%)	XX	XX (XX%)	XX
CT genotype: Serious adverse events, possibly, probably, or definitely related to the study drug, listed by type	XX (XX%)	XX	XX (XX%)	XX
TT genotype: Any serious adverse event possibly, probably, or definitely related to the study drug	XX (XX%)	XX		
TT genotype: Serious adverse events, possibly, probably, or definitely related to the study drug, listed by type	XX (XX%)	XX		

All TT genotype participants received dexamethasone. N.pt = the number of patients with at least one event (% of all patients receiving the same intervention). N.ae = the total number of episodes of that particular event.

**Table 15.** Summary of adverse events leading to TB treatment interruptions.

	Dexamethasone		Placebo	
Characteristic	(N=…)		(N=…)	
	N.pt	N.ae	N.pt	N.ae
CC genotype: Any adverse event leading to TB treatment interruption	XX (XX%)	XX	XX (XX%)	XX
CC genotype: Any adverse event leading to TB treatment interruption, listed by type	XX (XX%)	XX	XX (XX%)	XX
CT genotype: Any adverse event leading to TB treatment interruption	XX (XX%)	XX	XX (XX%)	XX
CT genotype: Any adverse event leading to TB treatment interruption, listed by type	XX (XX%)	XX	XX (XX%)	XX
TT genotype: Any adverse event leading to TB treatment interruption	XX (XX%)	XX		
TT genotype: Any adverse event leading to TB treatment interruption, listed by type	XX (XX%)	XX		

All TT genotype participants received dexamethasone. N.pt = the number of patients with at least one event (% of all patients receiving the same intervention). N.ae = the total number of episodes of that particular event.

**Table 16.** Summary of Grade 3&4 laboratory abnormalities.

	Dexamethasone		Placebo	
Characteristic	(N=…)		(N=…)	
	N.pt	N.ae	N.pt	N.ae
CC genotype: Any grade 3&4 laboratory abnormality	XX (XX%)	XX	XX (XX%)	XX
CC genotype: Grade 3&4 laboratory abnormalities, listed by type	XX (XX%)	XX	XX (XX%)	XX
CT genotype: Any grade 3&4 laboratory abnormality	XX (XX%)	XX	XX (XX%)	XX
CT genotype: Grade 3&4 laboratory abnormalities, listed by type	XX (XX%)	XX	XX (XX%)	XX
TT genotype: Any grade 3&4 laboratory abnormality	XX (XX%)	XX		
TT genotype: Grade 3&4 laboratory abnormalities, listed by type	XX (XX%)	XX		

All TT genotype participants received dexamethasone. N.pt = the number of patients with at least one event (% of all patients receiving the same intervention). N.ae = the total number of episodes of that particular event.

## Analysis of TT genotype participants

The following analyses will compare non-randomised TT genotype participants with CC and with CT participants, randomised to either dexamethasone or placebo, as follows:

A. All-cause mortality or new neurological event over the first 12 months after randomisation, comparison based on Kaplan-Meier estimates and log-rank test

- TT vs CTdex vs CCdex
- TT vs CTplacebo vs CCplacebo

B. Death over the first 12 months after randomisation, comparison based on Kaplan-Meier estimates and log-rank test

- TT vs CTdex vs CCdex
- TT vs CTplacebo vs CCplacebo

C. Rankin score ≥3 at 12 months after randomisation; proportions are compared

- TT vs CTdex vs CCdex
- TT vs CTplacebo vs CCplacebo

D. Measurements of blood and CSF inflammation (analyses same as for secondary outcome 6)

- *At baseline (blood transcriptomic and CSF Olink):*
  ○ TT vs CT vs CC
- *At day 14 and day 60 (blood transcriptomic data)*
  ○ TT vs CCdex vs CTdex
  ○ TT vs CCplacebo vs CTplacebo
- *At day 30 (CSF Olink data)*
  ○ TT vs CCdex vs CTdex
  ○ TT vs CCplacebo vs CTplacebo

## Meta-analysis

To investigate the benefit of dexamethasone in all adults with TBM, regardless of *LTA4H* status, we will conduct an individual patient data meta-analysis and combine the current trial data with data from 447 HIV-negative adults with TBM enrolled into a previous trial of adjunctive dexamethasone conducted in Vietnam ^4^. Baseline characteristics (age, sex, enrolment MRC TBM grade, diagnostic category) will be compared between trials and presented in a table. The 2004 trial was conducted prior to the creation of the Marais diagnostic criteria, therefore there are incomplete information to apply the criteria. We will therefore categorise participants into either definite (microbiologically confirmed), or combined probable or possible TBM. The primary outcome will be death within 9 months from randomisation. The secondary outcome will be death or disability (Rankin score ≥3) at 9 months from randomisation. Individuals who withdrew or were lost to follow-up before 9 months are excluded. For this 9-month assessment an acceptable range of -10 days/+1 month will be applied. The meta-analysis for survival will be quantified via the hazard ratio (Cox model) and the RMTL, and visualised using Kaplan-Meier plots. The secondary analysis is a test for difference in proportion. No correction for MRC grade will be made.

## Ethics and consent

The trial received ethics approval from the Oxford Tropical Research Ethics Committee (approval number 52-16, initial approval date 27/01/2017), the Ethics Committees of the Hospital for Tropical Diseases (approval number CS/ND/17/27, initial approval date 25/09/2017) and Pham Ngoc Thach Hospital (approval number CS/PT/17/06, initial approval date 08/09/2017), and the Vietnam Ministry of Health (approval number 5876/QĐ-BYT, initial approval date 29/12/2017). All participants provided written informed consent to enter the study.

## Data Availability

No data are associated with this article.
