# Supplementary material for: Statistical analysis plan for the LAST ACT clinical trial; a Leukotriene A4 hydrolase Stratified non-inferiority Trial of Adjunctive Corticosteroids for HIV-negative adults with Tuberculous meningitis
Source: Wellcome Open Res. 2025 Feb 5;9:695. Originally published 2024 Nov 27. [Version 2] doi: 10.12688/wellcomeopenres.22498.2 (PMC11795021; doi:10.12688/wellcomeopenres.22498.2)
Supplement: Peer review history

# Reviewer report: Reviewer response for version 2

**DOI:** 10.21956/wellcomeopenres.26233.r118211

**Published:** 2025-02-13

**Authors:** Michael Thy

**Affiliations:** 1 Université Paris Cité, Paris, France; 2 Infectious diseases and medical ICU, Hospital Bichat - Claude-Bernard, Paris, Île-de-France, France

Dear authors

 Thank you for the answers.

 Best regards

Is the study design appropriate for the research question?

Yes

Is the rationale for, and objectives of, the study clearly described?

Yes

Are sufficient details of the methods provided to allow replication by others?

Yes

Are the datasets clearly presented in a useable and accessible format?

Partly

Reviewer Expertise:

infectiologist and intensivist, topic in neurological tuberculosis

I confirm that I have read this submission and believe that I have an appropriate level of expertise to confirm that it is of an acceptable scientific standard.

# Reviewer report: Reviewer response for version 1

**DOI:** 10.21956/wellcomeopenres.24790.r117077

**Published:** 2025-02-04

**Authors:** Fajri Gafar

**Affiliations:** 1 Unit of PharmacoTherapy, -Epidemiology and -Economics, Groningen Research Institute of Pharmacy, University of Groningen, Groningen, The Netherlands

This is an interesting manuscript submitted by Joseph Donovan on the statistical analysis plan for the LTA4H-stratified trial of adjunctive corticosteroids for HIV-negative adults with TBM. The manuscript is well-written, informative, and clearly describes the planned analyses for the primary and secondary endpoints of the trial, which will provide important insights into the benefits (or harms) of corticosteroids in HIV-negative adults with TBM who have the CC or CT genotype.

Is the study design appropriate for the research question?

Yes

Is the rationale for, and objectives of, the study clearly described?

Yes

Are sufficient details of the methods provided to allow replication by others?

Yes

Are the datasets clearly presented in a useable and accessible format?

Not applicable

Reviewer Expertise:

Pharmacotherapy, clinical pharmacology, PK/PD

I confirm that I have read this submission and believe that I have an appropriate level of expertise to confirm that it is of an acceptable scientific standard.

# Reviewer report: Reviewer response for version 1

**DOI:** 10.21956/wellcomeopenres.24790.r116763

**Published:** 2025-01-21

**Authors:** Michael Thy

**Affiliations:** 1 Université Paris Cité, Paris, France; 2 Infectious diseases and medical ICU, Hospital Bichat - Claude-Bernard, Paris, Île-de-France, France

Dear Authors,

 Thank you for these interesting planned analyses for a Leukotriene A4 hydrolase Stratified Trial of Adjunctive Corticosteroids for HIV-negative adults with Tuberculous meningitis.

 I have some comments :

 1) How to get the genotype TT before treatment ?

 The technique for genotyping should be detailed, specifically the delay to obtain the results and what happen if the result is not available yet, is the patient secondarily excluded or included for ITT study ?

 in the LAST ACT protocol, it is written that "Randomisation will occur once the result of the LTA4H genotyping is available."

 2) The description of radiological findings should be taken into account at least in the description. e.g. a tuberculoma according to his size, with or without oedema, may have a different response than a arachnoiditis, etc.

 -Ideally for further analysis for correlation between the genotype (and inflammation biomarkers) and the radiological/clinical presentation.

 3) The rationale for TT genotype steroids response should be explicited and at least add more references supporting it.

 The actual ref seems the wrong one from Tobin et al. 2010 one and not the 2012 one.

 Maybe some data could be added :

 -steroids dependence ?

 -the use of TNFalpha inhibitors ?

 Thank you

 Best regards

Is the study design appropriate for the research question?

Yes

Is the rationale for, and objectives of, the study clearly described?

Yes

Are sufficient details of the methods provided to allow replication by others?

Yes

Are the datasets clearly presented in a useable and accessible format?

Partly

Reviewer Expertise:

infectiologist and intensivist, topic in neurological tuberculosis

I confirm that I have read this submission and believe that I have an appropriate level of expertise to confirm that it is of an acceptable scientific standard, however I have significant reservations, as outlined above.

## References

[1] : Host genotype-specific therapies can optimize the inflammatory response to mycobacterial infections. *Cell* .2012;148(3) : 10.1016/j.cell.2011.12.023 434-46 10.1016/j.cell.2011.12.023 22304914 PMC3433720

# Response

**Published:** 2025-02-04

**Authors:** Joseph Donovan

**Affiliations:** Oxford University Clinical Research Unit, Ho Chi Min City, Vietnam

Dear Dr Thy,

 Thank you for your review of our manuscript “Statistical analysis plan for the LAST ACT clinical trial; a Leukotriene A4 hydrolase Stratified non-inferiority Trial of Adjunctive Corticosteroids for HIV-negative adults with Tuberculous meningitis” Our responses to your comments are below:

*Question 1) How to get the genotype TT before treatment ?*

*The technique for genotyping should be detailed, specifically the delay to obtain the results and what happen if the result is not available yet, is the patient secondarily excluded or included for ITT study ?*

*in the LAST ACT protocol, it is written that "Randomisation will occur once the result of the LTA4H genotyping is available."*

**Response 1. ** The *LTA4H *rs17525495 (C/T) polymorphism was genotyped using the TaqMan genotyping assay, and then confirmed by sequencing. We have added this detail to the statistical analysis plan and given a reference where more detail is provided. *LTA4H* genotyping is performed daily in the OUCRU laboratories except for weekends. The lack of availability of this test at the weekend accounts for any delays. A participant will not commence study drug until the *LTA4H* genotype result is available (whether randomised to dexamethasone or placebo for CC or CT genotypes, or allocated to open dexamethasone for TT genotype). If, whilst waiting for the *LTA4H* result to return, an event develops which means a participant is no longer eligible for randomisation (or no longer eligible to start dexamethasone if the *LTA4H* genotype returns as TT) then this participant is not randomised (or will not start dexamethasone if TT). Such participants will not be included in the intention-to-treat analysis population. 

*Question 2) The description of radiological findings should be taken into account at least in the description. e.g. a tuberculoma according to his size, with or without oedema, may have a different response than a arachnoiditis, etc.*

*-Ideally for further analysis for correlation between the genotype (and inflammation biomarkers) and the radiological/clinical presentation.*

**Response 2. ** Radiological findings will be described in detail in a subsequent sub study. In an MRI sub study, radiological findings will be correlated with *LTA4H* genotype, and response to dexamethasone.  

*Question 3) The rationale for TT genotype steroids response should be explicited and at least add more references supporting it.*

*The actual ref seems the wrong one from Tobin et al. 2010 one and not the 2012 one.*

*Maybe some data could be added :*

*-steroids dependence ?*

*-the use of TNFalpha inhibitors ?*

**Response 3.** The rationale supporting use of corticosteroids in individuals of TT genotype is described in detail in the trial protocol (Wellcome Open Res. 2018 Mar 20:3:32). We have now added a second reference, that describes the retrospective *LTA4H* testing of 182 Vietnamese adults without HIV co-infection, with microbiologically confirmed TB meningitis.(this is the Tobin 2012 reference; Cell. 2012 Feb 3;148(3):434-46) Adrenal responsiveness following corticosteroid use will be described in a sub study analysis (described in more detail in the study protocol). TNF-alpha inhibitors were not used in this study.****

# Reviewer report: Reviewer response for version 1

**DOI:** 10.21956/wellcomeopenres.24790.r117075

**Published:** 2025-01-18

**Authors:** Ryan Dinkele

**Affiliations:** 1 University of Cape Town, Cape Town, South Africa

This study protocol describes the planned analytical approach for data collected during the LAST ACT clinical trial. The purpose of this analysis is to assess whether corticosteroid adjunct therapy – the current standard of care in treating HIV-negative patients with tuberculosis meningitis (TBM) – is beneficial or harmful to individuals with various host leukotriene A4 hydrolase genotypes, specifically CC, CT, and TT. While data exist to support the use of adjunct corticosteroids in the treatment of TBM patients with the TT genotype (a high-inflammatory phenotype), this research will provide important information about the harms or benefits for individuals with CT or CC genotypes, characterised by intermediate- and low-inflammatory phenotypes, respectively.

 This study protocol is well-written and informative, extensively detailing the analytical methods to be applied to the LAST ACT clinical trial data. The trial endpoints are clearly defined, and these analyses should yield valuable insights into dexamethasone use as an adjunct in TBM treatment.

Is the study design appropriate for the research question?

Yes

Is the rationale for, and objectives of, the study clearly described?

Yes

Are sufficient details of the methods provided to allow replication by others?

Yes

Are the datasets clearly presented in a useable and accessible format?

Not applicable

Reviewer Expertise:

Mycobacterium tuberculosis aerobiology, epidemiology, biostatistics.

I confirm that I have read this submission and believe that I have an appropriate level of expertise to confirm that it is of an acceptable scientific standard.
